# Everybody's Page

**Published:** 1890-06-07

**Authors:** 


### Everybody's Page.

During the year 1889, 65,379 van-loads of sweepings and refuse were removed by the scavengers from the City of London; of these loads many were removed from Letts' wharf, and nearly 16,000 loads of rubbish were burnt.

---

The total extinction of the Solomon islander in the very near future seems to be a certainty, now that traders have helped them to their doom by supplying them with Snider rifles. Always in a state of chronic warfare they can now indulge to their hearts' content in the wholesale destruction of entire villages.

---

The Governor General of Siberia is evidently a refined and humane gentleman. According to the Swiss paper the *Croix Federale*, he was so enraged recently at one prisoner, a woman, not rising on his entry to the prison at Tara, owing to the fact of her being in the last stage of consumption, that he ordered her to be sent at once to Verknioudinsk where she would receive much harsher treatment. In the eyes of this humane gentleman the illness of the wretched woman was an aggravation of her offence. Her death before she reached Verknioudinsk saved her from further insult and brutality. It is stated that political prisoners are henceforth to be treated still more harshly. Is there any treatment sufficiently harsh, any retribution sufficiently bitter for these wretched torturers who are lower than the brute creation and whose existence is a curse to mankind!

Mons. Vigerie's experiments as to the best methods to be adopted for the purpose of disinfection in the French army have led him to the conclusion that sulphuric acid should be discontinued as unreliable. For certain purposes bichlorate of mercury and steam commend themselves to him as being the most efficacious disinfectants.

---

Venice is not the only place which has boasted a "Libro d' Oro." We, here in London, if our pockets permit and our hearts are willing, can write our name in a golden book of kindness to little children. The office of "The Invalid Children's Aid Association" in Buckingham Street, is where the opportunity is to be found. Those little children who have been treated in the hospitals for spine, hip, or other diseases, and who only want kind care and nursing, or some simple surgical appliance, to bring them through their convalescence to perfect health, are looked after by this society; they put a finishing touch as it were to the good work begun by the hospitals. Sometimes very heavy cases must be helped, perhaps by a prolonged stay by the sea, entailing a cost of 4s. to 8s. a-week. Now is the time for the book to be searched; in its pages are written the names of those on whom the society can call for help in such an emergency, and who have promised to pay for a heavy case till recovery is complete. The golden book is not nearly filled, and those cases are very frequent. Who will inscribe their names?

